# Retrospective Analysis of Corticosteroid Treatment in Stevens-Johnson Syndrome and/or Toxic Epidermal Necrolysis over a Period of 10 Years in Vajira Hospital, Navamindradhiraj University, Bangkok

**DOI:** 10.1155/2014/237821

**Published:** 2014-06-15

**Authors:** Wanjarus Roongpisuthipong, Sirikarn Prompongsa, Theerawut Klangjareonchai

**Affiliations:** ^1^Division of Dermatology, Department of Medicine Vajira Hospital, Navamindradhiraj University, Bangkok 10300, Thailand; ^2^Research Center, Navamindradhiraj University, Bangkok 10300, Thailand; ^3^Department of Medicine, Faculty of Medicine, Ramathibodi Hospital, Mahidol University, Bangkok 10400, Thailand

## Abstract

*Background*. Stevens-Johnson syndrome (SJS) and/or toxic epidermal necrolysis (TEN) are uncommon and life-threatening drug reaction associated with a high morbidity and mortality. *Objective*. We studied SJS and/or TEN by conducting a retrospective analysis of 87 patients treated during a 10-year period. *Methods*. We conducted a retrospective review of the records of all patients with a diagnosis of SJS and/or TEN based on clinical features and histological confirmation of SJS and/or TEN was not available at the Department of Medicine, Vajira hospital, Bangkok, Thailand. The data were collected from two groups from 2003 to 2007 and 2008 to 2012. *Results*. A total of 87 cases of SJS and/or TEN were found, comprising 44 males and 43 females whose mean age was 46.5 years. The average length of stay was 17 days. Antibiotics, anticonvulsants, and allopurinol were the major culprit drugs in both groups. The mean SCORTEN on admission was 2.1 in first the group while 1.7 in second the group. From 2008 to 2012, thirty-nine patients (76.5%) were treated with corticosteroids while only eight patients (22.2%) were treated between 2003 and 2007. The mortality rate declined from 25% from the first group to 13.7% in the second group. Complications between first and second groups had no significant differences. *Conclusions*. Short-term corticosteroids may contribute to a reduced mortality rate in SJS and/or TEN without increasing secondary infection. Further well-designed studies are required to compare the effect of corticosteroids treatment for SJS and/or TEN.

## 1. Introduction

**Figure 1:**
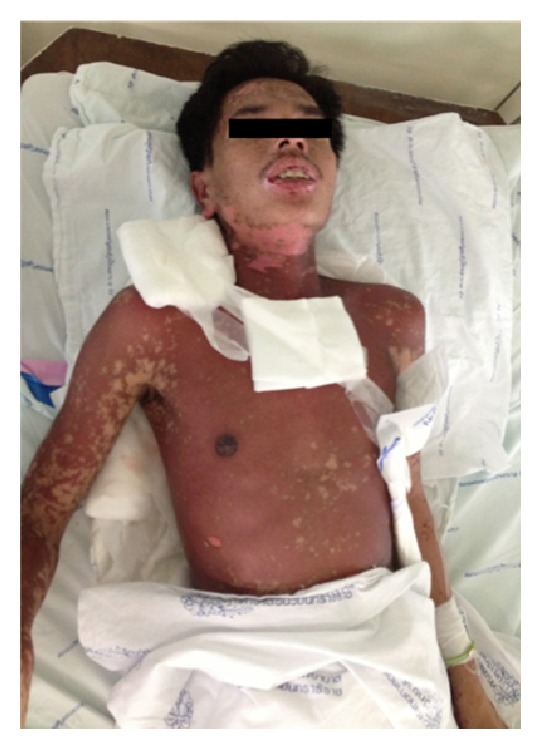
Multiple denuded areas on diffuse dusky red patches at forehead, neck, and right-sided trunk. Erosion on both upper and lower lips.

**Figure 2:**
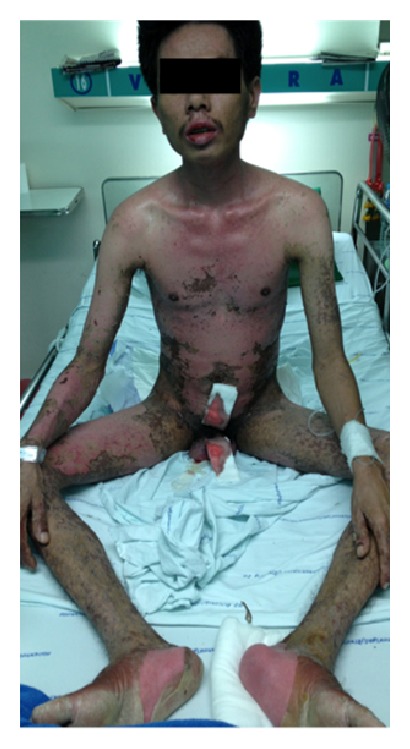
After 4 days, the patients developed progressive denuded area on previous dusky red. Patches on trunk and extremities. Erosion on both upper and lower lips, genitalia.

Steven-Johnson syndrome (SJS) and/or toxic epidermal necrolysis (TEN) are uncommon diseases with an incidence about 1.9 cases per million per year [1]. SJS and/or TEN are potentially mortal diseases, characterized by extensive blistering exanthema and epithelial sloughing, occurring with mucosal involvement (Figures 1 and 2) [2]. SJS and/or TEN are part of a spectrum, which is divided into 3 groups: SJS when the total detachment is less than 10% of the body surface area; TEN when it is over 30%; SJS-TEN overlap when it is between 10% and 30% [3]. Differential diagnoses of SJS and/or TEN are linear IgA bullous disease, paraneoplastic pemphigus, generalized bullous fixed drug eruption, and staphylococcal scalded skin syndrome. Even though many factors have been proposed as causes of these diseases, hypersensitivity to medications reports for the most of cases. *β*-lactam antibiotics, sulfonamides, anticonvulsants, and allopurinol were frequent triggers of SJS and/or TEN [4]. The SCORTEN indicates a severity of illness, which is strongly correlated with the risk of death [5]. Aside from intensive supportive treatment, a normally accepted regimen for specific therapy of SJS and/or TEN is lacking. Treatment options include systemic corticosteroids, intravenous immunoglobulin therapy (IVIG), thalidomide, and TNF-*α* antagonist. Traditionally systemic corticosteroids were advocated until early 1990s, although no benefit has been demonstrated in case-controlled studies [6]. A retrospective single center study proposes that short-term dexamethasone therapy, given at an early stage of the disease, may contribute to a reduced mortality rate [7]. Moreover, the study from a general hospital in Singapore reports that the use of dexamethasone therapy may be a benefit [8]. The argument over systemic corticosteroid usage will still be continuously unresolved. The aim of this study was to present the etiologies, treatment, and clinical outcomes of SJS and/or TEN in Vajira Hospital, Navamindradhiraj University in Bangkok, Thailand.

## 2. Methods

A retrospective review was performed on patients admitted to Vajira Hospital, Navamindradhiraj University, with the diagnosis of SJS and/or TEN based on clinical features and histological confirmation of SJS and/or TEN was not available. The data were collected into two groups from 2003 to 2007 and 2008 to 2012 (10-year study). The ethical review board of the Faculty of Medicine Vajira Hospital, Navamindradhiraj University, approved this study.

The electronic medical database and inpatient charts were reviewed. The following data were collected: demographic information, culprit drugs, extent of mucocutaneous involvement, underlying diseases, laboratory data, treatments, complications, and mortality. Drugs that have been taken within 6 weeks before the onset of symptoms were considered as culprit drugs. If the patient had taken more than one drug, all of them were considered as culprit drugs.

## 3. Statistical Analysis

Continuous variables are reported as mean ± SD and data for categorical variables are reported as numbers and percentages. Comparisons of categorical variables among groups were performed using *χ* ^2^ test or Fisher's test. Comparisons of continuous variables among groups were performed using unpaired Student's *t*-test or Mann-Whitney *U* test. Statistical significance was set at *P* < 0.05 (two-tailed). Statistical analysis was performed with the SPSS version 18.0 (SPSS Inc., Chicago, IL, USA).

## 4. Results

Eighty-seven patients (44 males and 43 females) were admitted during this period. There were 36 cases (mean age was 42.6) since the year of 2003 until 2007 and 51 cases (mean age was 49.3) since the year of 2008 until 2012. In the first group, 36 cases were classified as SJS 26 cases (70.6%), SJS-TEN overlap 1 cases (2.8%), and TEN 9 cases (25.0%). In the second group, 51 cases were classified as SJS 36 cases (70.6%), SJS-TEN overlap 7 cases (13.7%), and TEN 8 cases (15.7%). Cardiovascular disease, diabetes mellitus, and HIV infection were not different between the first and second groups. Malignancy was 7 cases (13.7%) in the second group, while there was no case of malignancy in the first group. Mucosal involvement involved mouth more than other sites in both groups. Urethral involvement in the first group was significantly higher than the second group, while genital involvement in the second group was significantly higher than the first group. The mean of SCORTEN on the day of admission was 1.7 in the first group and 2.1 in the second group. In the second group, thirty-nine patients (76.5%) were treated with intravenous corticosteroids; the most common agent was dexamethasone. Only eight patients (22.2%) were treated with intravenous corticosteroid in the first group. The duration and dose of corticosteroid did not differ between the two groups. No patient received intravenous immunoglobulin.  Table 1 shows clinical characteristics for the 87 patients.  Table 2 shows percentage of intravenous steroid usage in SJS and/or TEN patients stratified by SCORTEN.

All of the patients in this study were related to drug administration. Antibiotics, anticonvulsants, and allopurinol were the major culprit drugs in both groups (Table 3). The highest culprit drugs were allopurinol (19.1%) in the first group and phenytoin (13.8%) in the second group. Penicillin and cotrimoxazole were the most frequent among antibiotics and phenytoin was the most frequent among anticonvulsants in both groups.

Many patients showed organ involvement and other complications (Table 4). Respiratory failure was the most internal organ failure in both groups. Endotracheal intubation and mechanical ventilation were needed for all of these patients. Liver and renal dysfunctions were more common in the first group than in the second group. Sepsis was more in the first group than in the second group, while skin infection and hospital-acquired pneumonia were more in the second group than in the first group. The admission duration was average 13.9 days in the first group and 19.2 days in the second group. The mortality rate declined from 25% from the first group to 13.7% in the second group.

## 5. Discussion

In our study, incidence of SJS and/or TEN was 8-9 cases per year which is similar to another report from Asia such as Thailand and Korea [9, 10]. The mean age was approximately 46 years which is as high as those reported from other countries in Asia such as Japan, Singapore, and Korea [2, 8, 10]. In contrast to earlier studies showing that females are affected with SJS and/or TEN more than males [2, 10], our series had equal numbers of males and females, which was in agreement with the study done by Tan and Tay [8]. The most common culprit drug group in this study was antibiotics (penicillin group and sulfonamide group) similar to other studies in Thailand [9, 11] and other Asian countries [2, 12]. Allopurinol showed a higher risk in this study than in previous studies [2, 9, 10]. It was the most common culprit drugs similar to EuroSCAR study [13]. The incidence of allopurinol associated with SJS or TEN increased in the EuroSCAR study because of increasing usages and dosages of this drug. This study revealed that the incidence of allopurinol associated with SJS or TEN declined from 19% in the first group to 12% in the second group. It may be hypothesized that the decreased rate is associated with physician's caution use allopurinol to accepted guidelines and adjusted dosage base on renal function. Carbapenems, a board spectrum of antibiotics, are increasingly used in clinical practice [14]. In this study, carbapenem-associated SJS or TEN was reported to be 3.4% between 2008 and 2012. In addition, Carbapenems are *β*-lactam; therefore, they can cross-react with penicillins or cephalosporins. There was a report of two successive episodes of cephalosporin and carbapenem associated with TEN in the same patient; therefore, drug having chemical similarity to the initial causative compound should be strictly avoided in management of SJS or TEN [15].

Management in SJS or TEN involves sequentially rapid evaluation of the severity and prognosis of disease by using SCORTEN, prompting identification and discontinuation of all causative drugs, and initiating supportive care (such as fluid, electrolyte, wound, and nutritional management) and eventual specific treatment. Up till now, a specific treatment for SJS or TEN that has shown efficacy in controlled trials does not exist. The use of systemic corticosteroids in SJS or TEN is controversial. Although corticosteroids have pleomorphic immunomodulating effect through inhibition of various cytokines, the use of corticosteroids and prolong use of corticosteroids increase the risk of secondary infection and masking early sign of sepsis. Therefore, the use of corticosteroids is usually limited in SJS or TEN. In the present study, the use of systemic corticosteroids increased from 22% in the first group to 76% in the second group. Moreover, corticosteroid treatment duration for more than 7 days declined from 50% in the first group to 33% in the second group. In the second group, mortality and sepsis significantly declined when compared to the first group, while rate of hospital-acquired pneumonia and skin infection did not change. Additionally, the first group had lower SCORTEN than the second group but the mortality rate was higher in the first group than in the second group. In interpreting these results, short-course systemic corticosteroids such as dexamethasone in SJS or TEN reveals the benefit of decreasing the mortality rate while not increasing secondary infection such as septicemia, respiratory tract, and skin infection. In addition, two monocenter retrospective studies suggested that short-course high-dose corticosteroids (dexamethasone) might be of benefit [7, 8]. On the other hand, a retrospective case-control study conducted in France and Germany concluded that corticosteroids did not show a significant effect on mortality in comparison with supportive care only [6]. A retrospective analysis had some pitfalls; therefore, multicentre, randomized, placebo-controlled trials using standardized design are required in order to investigate further the use of corticosteroid in SJS and/or TEN. In addition, such a system might be useful for evaluation of genetic marker.

## 6. Conclusions

The most common drug-related SJS and/or TEN in Vajira hospital was allopurinol and the most common drug group was antibiotics. Short-term corticosteroids may contribute to a reduced mortality rate in SJS and/or TEN without increasing secondary infection. Further well-designed studies are required to compare the effect of corticosteroids treatment for SJS and/or TEN.

## Figures and Tables

**Table 1:** Clinical characteristics of Stevens-Johnson syndrome and/or toxic epidermal necrolysis cases from 2003 to 2012 (*n* = 87).

	2008–2012 (*n* = 51), *n* (%)	2003–2007 (*n* = 36), *n* (%)	*P* value
Age (years)	49.3 ± 19.2	42.6 ± 21.0	0.104
Male	27 (52.9)	17 (47.2)	0.599
Underlying diseases			
Cardiovascular disease	11 (21.5)	8 (22.2)	0.942
Diabetes mellitus	7 (13.7)	5 (13.8)	0.983
HIV infection	12 (23.5)	9 (25.0)	0.875
Malignancy∗	7 (13.7)	0 (0)	0.033
Diagnosis			
SJS	36 (70.6)	26 (72.2)	0.868
SJS-TEN overlap	7 (13.7)	1 (2.8)	0.082
TEN	8 (15.7)	9 (25.0)	0.281
Mucosal involvement			
Ocular	40 (78.4)	32 (88.8)	0.203
Mouth	45 (88.2)	35 (97.2)	0.129
Genitalia∗	27 (52.9)	11 (30.5)	0.038
Urethra∗	2 (3.9)	7 (19.4)	0.019
Anus	3 (5.8)	1 (2.8)	0.496
SCORTEN			
≤1	16 (31.4)	13 (36.1)	0.664
2	19 (37.3)	19 (52.8)	0.151
3	12 (23.5)	3 (8.3)	0.065
4	1 (1.9)	1 (2.8)	0.802
≥5	3 (5.8)	0 (0)	0.139
Causes of disease			
Single drug-related	44 (86.3)	30 (83.3)	0.705
Multiple drug-related	7 (13.7)	6 (16.6)	0.705
Intravenous steroid use∗∗	39 (76.5)	8 (22.2)	<0.001
Dexamethasone equivalent doses (mg/day)	13.2 ± 6.1	14.5 ± 6.3	0.914
Steroid treatment duration (day)	5.7 ± 2.7	5.4 ± 2.5	0.810
Steroid treatment duration of ≥7 days	13 (33.3)	4 (50.0)	0.096

*P < 0.05,**P < 0.01.

SJS: Stevens-Johnson syndrome.

TEN: toxic epidermal necrolysis.

**Table 2:** Percentage of intravenous steroid usage in Stevens-Johnson syndrome and/or toxic epidermal necrolysis patients stratified by SCORTEN.

	2008–2012 (*n* = 51)	2003–2007 (*n* = 36)
SCORTEN		
≤1	87.5%	15.4%
2	63.1%	13.0%
3	83.3%	33.3%
4	100%	0%
≥5	66.7%	—

**Table 3:** Comparison of incidences of culprit drugs.

	2008–2012 (*n* = 58), *n* (%)	2003–2007 (*n* = 42), *n* (%)	*P* value
Antibiotics	26 (44.8)	14 (33.3)	0.265
Penicillin	7 (12.1)	4 (9.5)	0.718
Cotrimoxazole	7 (12.1)	4 (9.5)	0.718
Cephalosporin	5 (8.6)	2 (4.8)	0.473
Quinolone	3 (5.2)	2 (4.8)	0.949
Carbapenem	2 (3.4)	0 (0)	0.229
Clindamycin	1 (1.7)	0 (0)	0.398
Tetracycline	1 (1.7)	0 (0)	0.398
Macrolide	0 (0)	2 (4.8)	0.089
Anticonvulsants	14 (24.1)	4 (9.5)	0.064
Phenytoin	8 (13.8)	3 (7.1)	0.309
Carbamazepine	4 (6.9)	1 (2.4)	0.317
Phenobarbital	1 (1.7)	0 (0)	0.398
Lamotrigine	1 (1.7)	0 (0)	0.398
Allopurinol	7 (12.1)	8 (19.1)	0.301
NSAIDs	5 (8.6)	4 (9.5)	0.844
Nevirapine	3 (5.2)	4 (9.5)	0.377
Antituberculosis^a^	3 (5.2)	0 (0)	0.139
Other drugs	0 (0)	8 (19.1)	
TTM	0 (0)	2 (4.8)	0.089
Valacyclovir	0 (0)	2 (4.8)	0.089
Cetirizine	0 (0)	1 (2.4)	0.231
Chloroquine	0 (0)	1 (2.4)	0.231
Cinnarizine	0 (0)	1 (2.4)	0.231
Silymarin	0 (0)	1 (2.4)	0.231

^a^Antituberculosis (isoniazid, rifampicin, pyrazinamide, and ethambutol).

NSAIDs: nonsteroidal anti-inflammatory drugs.

TTM: traditional Thai medicine.

**Table 4:** Organ involvement and complications in patient with Stevens-Johnson syndrome and/or toxic epidermal necrolysis cases from 2003 to 2012 (*n* = 87).

	2008–2012 (*n* = 51), *n* (%)	2003–2007 (*n* = 36), *n* (%)	*P* value
Internal organ involvement			
Liver failure	3 (5.9)	3 (8.3)	0.657
Renal failure	6 (11.8)	6 (16.6)	0.514
On hemodialysis	3 (5.9)	3 (8.3)	0.657
Respiratory failure			
On ventilator	7 (13.7)	6 (16.6)	0.705
Infections			
Skin infection	9 (17.3)	6 (16.6)	0.905
Hospital-acquired pneumonia	7 (13.7)	4 (11.1)	0.718
Sepsis	7 (13.7)	8 (22.2)	0.301
Length of stay	19.2 ± 15.8	13.9 ± 9.6	0.287
Death	7 (13.7)	9 (25)	0.181
